# Could negative outcomes of psychotherapies be contributing to the lack of an overall population effect from the Australian Better Access initiative?

**DOI:** 10.1177/10398562231172417

**Published:** 2023-04-25

**Authors:** Stephen Allison, Jeffrey CL Looi, Steve Kisely, Tarun Bastiampillai

**Affiliations:** College of Medicine and Public Health, 1065 Flinders University of South Australia, Adelaide, SA, Australia; and; Consortium of Australian-Academic Psychiatrists for Independent Policy and Research Analysis (CAPIPRA), Canberra, ACT, Australia; Academic Unit of Psychiatry and Addiction Medicine and Addiction Medicine, 2219 The Australian National University School of Medicine and Psychology, Canberra, ACT, Australia; and; Consortium of Australian Academic Psychiatrists for Independent Policy Research and Analysis, Canberra, ACT, Australia; School of Medicine, 1974 University of Queensland, Woolloongabba, QLD, Australia; Psychiatry, 1065 Flinders University, Adelaide, SA, Australia; and; Psychiatry, Monash University, Clayton, VIC, Australia

**Keywords:** Psychotherapies, negative effects, Australian Medicare Better Access initiative

## Abstract

**Objective:**

We examine deterioration in psychotherapies, as reported in the recent evaluation of the Australian Medicare Better Access initiative.

**Conclusion:**

A focus on patients who experience poor clinical outcomes helps programs minimise harm and improve quality of care. The Better Access evaluation found the mental health of 20–40% of patients deteriorated. This may partly explain why population distress and suicide rates were not reduced by the introduction of the Better Access initiative. Deterioration was more likely for milder conditions, and less likely for severe conditions, which also improved the most. Using severity as a criterion for priority setting and resource allocation may minimise patient risk and maximise benefits. Patients with severe conditions may require considerably more sessions than the current average for Better Access psychotherapies.

To reduce the burden of common mental health conditions in the community, the Australian federal government has invested in psychotherapies through the Medicare Better Access initiative.^1^ By 2021, one in every 10 Australians received at least one Better Access service, and one in 20 had one or more sessions of psychotherapy, at a cost to the Australian government of AUD 1.2 billion.^2^ Despite this large investment, the prevalence of psychological distress and suicide were not reduced by the introduction of Better Access in 2006.^3^ This raises questions about why increased access to psychotherapies has not improved population mental health.^4^

## Deterioration in psychotherapies

Several possible reasons exist for the lack of an observed population effect from Better Access psychotherapies. Firstly, psychotherapies are only modestly efficacious for common mental health conditions. For example, the pill placebo response rate of depressive disorders is around 40%, and measured against this comparator, Cognitive Behavioural Therapy (CBT) has a number needed to treat (NNT) of 13, and antidepressants have similar efficacy (NNT = 9).^5^ These modest efficacies may be a reason why the mass rollout of first line treatments has not reduced the community prevalence of depression.^4^ CBT might be even less efficacious that the published trials suggest, if researchers ‘cherry pick’ findings that align with their views, and journals prefer to publish studies with positive results.^2^ In translating psychotherapies to the real world, CBT may have reduced effectiveness unless treatment is carefully targeted, expertly delivered, and of adequate duration.^4^ In 2021, the average number of psychotherapy sessions per patient in Better Access (5.4) was lower than most research trials of CBT.^1,2^

About a half of psychotherapy patients fail to improve or their mental health deteriorates in real world studies.^5^ Deterioration rates would be even higher in untreated populations, especially for severe conditions.^5^ Generally, deterioration is related to the underlying mental health condition or factors external to psychotherapy but is sometimes a side effect of psychotherapy with reports of unpleasant memories resurfacing, emergence of new symptoms, occupational problems, stigmatisation and self-blame, social network changes and strains in marital relationships.^6,7,8^ Adverse experiences of the therapeutic relationship include feeling violated by statements of the psychotherapist and inappropriate psychotherapist behaviour.^8,9^ If about 5–10% of patients deteriorate in psychotherapy, this could reduce the overall effectiveness of the Better Access iniatives at the population level.^6,9^

## Negative outcomes in the evaluation of Better Access

In December 2022, the Australian federal government released an independent evaluation of the Better Access iniative.^2^ Five of nine studies presented data on deterioration in psychotherapy (Table 1). Improvement rates were higher than deterioration rates in all these studies. Rates of deterioration varied widely from zero to 40%, with no significant change in zero to 30%, depending on the selected evaluation method.

**Table 1.** Significant change in the Better Access evaluation studies

Outcome study method	Significant deterioration (%)	No significant change (%)	Significant improvement (%)
Study 1: Clinical practice data (Care-episodes = 86,121)	10–20%	20–30%	50–60%
Study 2: Cross-sectional patient survey (*n* = 2013)	3%	6%	91%
Study 3: Observational prospective study (*n* = 2199)	20–30%	20–30%	45–55%
Study 4: Population level longitudinal studies (*n* = 30,906)	20–40%	20–30%	45–55%
Study 5: Patient interviews (*n* = 37)	0%	0%	100%

### Study 1

Routine clinical practice outcome data was used for a before-and-after study of 86,121 episodes of care delivered by more than 3000 private psychologists (Table 1). Females received about two thirds of the care, and 40–65% of the care-episodes involved those under 40 years. Referencing an effect size of 0.3 based on a range of standardised measures, episodes were divided into ‘significant improvement’, ‘no significant change’ or ‘significant deterioration’ (Table 1). Around 10–20% of the care-episodes were accompanied by deterioration, and 20–30% had no significant change. Milder episodes showed the most deterioration, and more severe episodes showed the least deterioration across a wide range of measures.

Pirkis et al. concluded that this study ‘provides evidence that Better Access is achieving positive outcomes for many consumers, particularly those who seek care when they are experiencing relatively severe depression, anxiety and/or psychological distress’.^2^ However, this is an uncontrolled study. Spontaneous improvement, social support, personal coping strategies, statistical regression-towards-the-mean, positive expectations, concomitant use of medications, along with Better Access psychotherapies are all potential components of these positive outcomes.^5^ Equally, deterioration during Better Access psychotherapies was likely due to a combination of factors related to treatment (e.g., short duration), and external factors.

### Study 2

This cross-sectional patient survey involved a large sample of over 2000 patients recruited through Medicare, but the overall response rate was low (7.4%). The sample comprised regular users of Better Access psychotherapies, with two thirds of the respondents having received Better Access treatment previously. Most were still receiving treatment at the time of the survey, and half were likely to attend more than 10 sessions. Only a small proportion (3%) reported significant deterioration (Table 1). The majority (91%) rated their mental health as improved. Most respondents were satisfied (41%) or very satisfied (45%) with psychological treatment. Since the survey used retrospective self-report, recall bias and social desirability bias might have affected these results.^2^ Pirkis et al. noted: ‘Participants may have had difficulty remembering what their mental health was like before and after their episode of mental health care, and may have been inclined to indicate that it was better after the episode’.^2^

A small proportion of the respondents (*n* = 224) had dropped out of Better Access treatment, providing insights into possible reasons for dissatisfaction. Some (38%) dropped out because the sessions were not helpful, 32% due to out-of-pocket costs and 30% did not like the mental health professional’s manner or approach. Only 28% indicated that they stopped Better Access treatment early because they felt better.

### Study 3

This observational prospective study (*n* = 2199) included two large-scale randomised controlled trials of primary mental health care. Standardised questionnaires were completed at set time points. This new analysis focused on a subset of the control groups who used Better Access services delivered by allied health professionals. About 20–30% of the patients deteriorated on measures of depression, anxiety, and total days out of role, while one-third experienced significant deterioration in quality of life (Table 1). Significant improvement was associated with poorer mental health at the start of the study. Pirkis et al. concluded: ‘This makes sense; those who begin treatment with severe mental health symptoms have had a greater window of opportunity for improvement’.^2^

### Study 4

This population-level study involved a new analysis of data from two large-scale Australian longitudinal studies (*n* = 30,906). Patients who used Better Access psychotherapies accessed a median of 5–6 sessions from private psychologists. The mental health of 45–55% of these patients improved significantly. Approximately, 20–40% had worse mental health on various measures, and 20–30% had no significant change (Table 1). Better baseline mental health predicted deterioration. Pirkis et al. commented: ‘it is worrying that some consumers experience deterioration in their mental health in not insignificant numbers of episodes, and that some show no change. These consumers are most likely to be people who began their episode with relatively mild symptoms or high levels of functioning or satisfaction with life’.^2^

### Study 5

Qualitative patient interviews were conducted with patients (*n* = 37) recruited through Beyond Blue and Lived Experience Australia. Patients reported overwhelmingly positive experiences with Better Access psychotherapies, that is, ‘All participants reported positive changes to their health and wellbeing since seeing their psychologist’.^2^ They felt more hopeful and empowered, mood improved, social confidence increased, and workforce participation was enhanced. Respondents praised the manner and approach of the psychologist as being the main reason for positive change. Major insights into potential reasons for deterioration or failure to respond to psychotherapies were not available.

## Policy implications of negative outcomes

The mental health of 20–40% of patients deteriorated in population level longitudinal studies of the Better Access initiative. Given that 1.3 million patients received Better Access psychological treatment in 2021, many patients may have experienced deterioration in their mental health, especially those with milder symptoms. This may have offset some of the potentially positive effects of the initiative. The survey and interview studies (2 and 5 in Table 1) found far lower rates of deterioration than the quantitative studies, raising concerns about the representativeness of these study populations.

The Better Access evaluation did not investigate the possible causes of deterioration in psychotherapy. Future evaluations could examine whether deterioration is a side effect of psychotherapies, due to sub-optimal delivery of CBT and/or factors external to psychotherapy. Quantitative studies could include specific questionnaires that measure side effects of psychotherapies.^7^ Qualitative studies could recruit patients who report deterioration, in order to explore consumer perspectives, and whether some patients attribute the worsening of their mental health to side effects of psychotherapy. Such studies would inform policymakers about the risks of Better Access psychotherapies with the aims of minimising harm and improving the program’s effectiveness.

In conclusion, this most recent Better Access evaluation reminds policymakers that psychotherapies are generally developed for clinical cohorts with more severe conditions. The mass rollout of brief psychotherapies for milder conditions does not appear to reduce population distress or suicide rates, and a considerable proportion of these patients experience deterioration.^2,3,4^ Offering treatment for milder symptoms might undermine personal coping abilities and social support networks.^9,10^ Deterioration was less likely for patients with severe symptoms, and they also experienced more improvement.^2^ Based on these findings, severity could be used as a criterion for priority setting and resource allocation. Instead of the mass rollout of brief psychotherapies for milder conditions, prioritising longer courses of psychotherapy for more severe conditions may minimise risk and maximise the potential benefits of the Better Access initiative.
